# Tris(1,1,1,5,5,5-hexa­fluoro-2,4-pentane­dionato-κ^2^ *O*,*O*′)molybdenum(III)

**DOI:** 10.1107/S1600536810005489

**Published:** 2010-02-13

**Authors:** Yohan Champouret, Rinaldo Poli, Jean-Claude Daran

**Affiliations:** a Laboratoire de Chimie de Coordination, CNRS UPR8241, 205 route de Narbonne, 31077 Toulouse cedex, France

## Abstract

In the title compound, [Mo(C_5_HF_6_O_2_)_3_], the unit cell is built up by three independent Mo^III^ atoms located on two different threefold axes. The three independent mol­ecules are roughly identical and each Mo^III^ atom is surrounded by three chelating hexa­fluoro­acetonate ligands in a three-bladed propeller-like arrangement, as observed in related compounds with acetyl­acetonate-type ligands. The structure of the title compound is very similar to the trigonal form of the Cr^III^ analogue. However, the latter crystallizes in a higher-symmetry space group, *P* 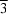 *c*1. Both crystals are twinned by merohedry with the same twin law (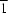 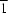
0/010/00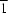) in reciprocal space, but the symmetry of the Laue group in which it operates is different, 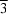 to 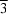 *m* for the title complex, and 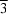 *m* to 6/*mmm* for the Cr^III^ complex.

## Related literature

For related Cr(hfac)_3 _structures (hfac is 1,1,1,5,5,5-hexa­fluoro­acetonate), see: Harada & Girolami (2007 ▶); Jessop *et al.* (2002 ▶). For a related Mo(acac)_3_ complex (acac is acetyl­acetonate), see: Raston & White (1979 ▶). For the synthetic procedure, see: Larsen & Sukup (1980 ▶).
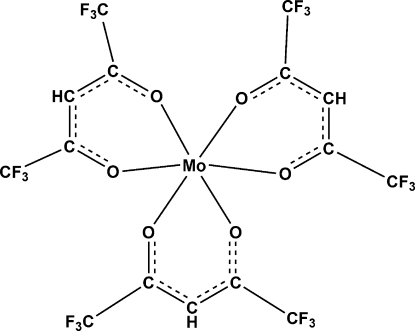


## Experimental

### 

#### Crystal data

[Mo(C_5_HF_6_O_2_)_3_]
*M* *_r_* = 717.11
Trigonal, 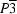

*a* = 18.4876 (10) Å
*c* = 11.5021 (7) Å
*V* = 3404.6 (3) Å^3^
*Z* = 6
Mo *K*α radiation
μ = 0.76 mm^−1^
*T* = 180 K
0.39 × 0.11 × 0.05 mm

#### Data collection

Oxford Diffraction XCALIBUR diffractometer
Absorption correction: multi-scan (*CrysAlis RED*; Oxford Diffraction, 2008 ▶) *T*_min_ = 0.731, *T*_max_ = 1.000
26629 measured reflections
4635 independent reflections
2801 reflections with *I* > 2σ(*I*)
*R*_int_ = 0.063

#### Refinement

*R*[*F* ^2^ > 2σ(*F* ^2^)] = 0.030
*wR*(*F* ^2^) = 0.068
*S* = 0.97
4635 reflections
362 parameters
H-atom parameters constrained
Δρ_max_ = 0.78 e Å^−3^
Δρ_min_ = −0.44 e Å^−3^

### 

Data collection: *CrysAlis CCD* (Oxford Diffraction, 2008 ▶); cell refinement: *CrysAlis RED* (Oxford Diffraction, 2008 ▶); data reduction: *CrysAlis RED*; program(s) used to solve structure: *SIR97* (Altomare *et al.*, 1999 ▶); program(s) used to refine structure: *SHELXL97* (Sheldrick, 2008 ▶); molecular graphics: *ORTEPIII* (Burnett & Johnson, 1996 ▶), *ORTEP-3 for Windows* (Farrugia, 1997 ▶) and *PLATON* (Spek, 2009 ▶); software used to prepare material for publication: *SHELXL97*.

## Supplementary Material

Crystal structure: contains datablocks I, global. DOI: 10.1107/S1600536810005489/gk2257sup1.cif

Structure factors: contains datablocks I. DOI: 10.1107/S1600536810005489/gk2257Isup2.hkl

Additional supplementary materials:  crystallographic information; 3D view; checkCIF report

## Figures and Tables

**Table 1:** Comparison of some geometric parameters (Å, °) within the chelating acetonate units of related compounds

Complex	*M*—O (mean value)	*M*—O—*M* (mean value)	O⋯O
[Mo(hfacac)_3_]^*a*^	2.053 (4)	87.01 (9)	2.829 (4)
[Mo(acac)]_3_^*b*^	2.072 (3)	87.4 (1)	2.866 (3)
[Cr(hfacac)_3_]^*c*^	1.957 (1)	90.63 (5)	2.782 (2)
[Cr(hfacac)_3_]^*d*^	1.949 (5)	90.8 (2)	2.776 (7)

Notes: (*a*) This work; (*b*) Raston & White (1979 ▶); (*c*) Jessop *et al.* (2002 ▶); (*d*) Harada & Girolami (2007 ▶). acac is acetyl­acetonate and hfacac is 1,1,1,5,5,5-hexa­fluoro­acetyl­acetonate.

**Fig. 1.**
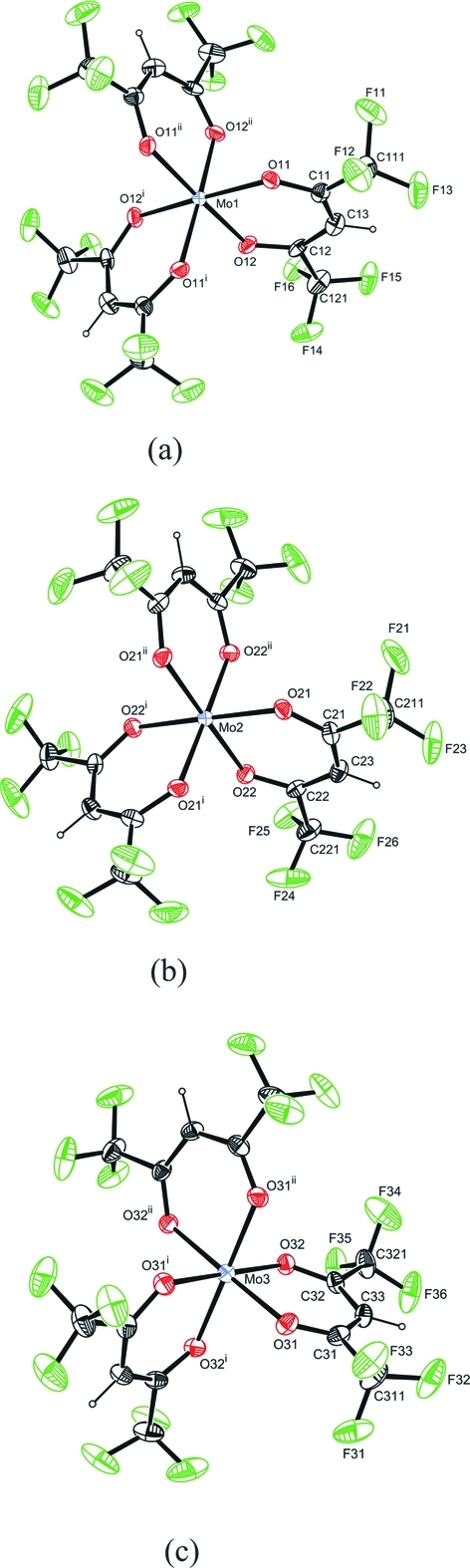
View of the three symmetry-independent molecules of (I) with atom labeling scheme. Dispalcement ellipsoids are shown at the 30% probability level. H atoms are represented as small spheres of arbitrary radii. (a) Molecule 1 [symmetry codes: (i) -y + 2, x - y + 1, z; (ii) -x + y + 1, -x + 2, z]; (b) molecule 2 [symmetry codes: (i) -x + y, -x + 1, z; (ii) -y + 1, x - y + 1, z]; (c) molecule 3 [symmetry codes: (i) -x + y, -x + 1, z; (ii) -y + 1, x - y + 1, z].

**Fig. 2.**
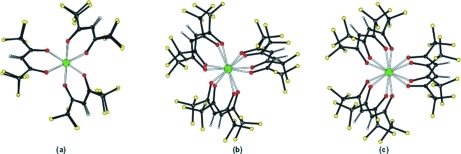
Molecular fitting of the three independent molecules: (a) molecular fitting of molecules 1 and 2; (b) molecular fitting of molecules 1 and 3; (c) molecular fitting of molecules 2 and 3.

## supplementary crystallographic information

### Comment

The title compound was of interest in our laboratory for potential application as reversible trapping agent of polymeric radical chains in controlled radical polymerization processes.

The stucture of the title compound, (I), is built up from three independent molybdenum centers located on two different three fold axes. As usually observed in such complexes (Raston & White, 1979; Jessop *et al.*, 2002; Harada & Girolami, 2007) each molybdenum is surrounded by three chelating hexafluoroacetylacetonate ligands in a three-bladed propellor-like arrangement (Fig.1). The three molecules are roughly identical as can be seen from the molecular fitting views (Fig. 2) realized using PLATON (Spek, 2009) , they only differ by slight change in orientation. The metal-O bonds and O—M—O bond angles within the chelating β-diketonate framework compare with related complexes (Table 1), the observed difference in M—O bond length being related to the diffrence in the covalent radii between Cr (1.39 (5) Å) and Mo (1.54 Å).

This compound appears to be isostructural with the related chromium complex (Harada & Girolami, 2007) although the space groups are different, P -3 for the title complex whereas it is reported as P -3c1 for the chromium complex. Both crystals are twinned by merohedry by the same twin law but the symmetry of the Laue group in which it operates is different: -3 to -3 m for the title complex whereas it is -3 m to 6/mmm for the Cr complex. The refinement of the title complex is much better than the one reported for the Cr complex (Harada & Girolami, 2007).

### Experimental

The compound was prepared by refluxing Mo(CO)_6_ in toluene in the presence of 3 equivalents of 1,1,1,5,5,5-hexafluoropentan-2,4-dione and a few drops of diglyme which is known to facilitate CO replacement reactions for the carbonyl molybdenum precursor. Single crystals of the product grew directly from the solution after cooling, concentration, filtration at room temperature, and further cooling to -20°C.

### Refinement

The crystal is twinned by merohedry with the -1 -1 0 0 1 0 0 0 -1 twin law in the reciprocal space. A scale factor for the major twin domain is 0.8076 (9).

The three H atoms attached to the central C atoms were fixed geometrically and treated as riding with C—H = 0.95 Å with U_iso_(H) = 1.2U_eq_(C).

### Crystal data

**Table:**

[Mo(C_5_HF_6_O_2_)_3_]	*D*_x_ = 2.099 Mg m^−^^3^
*M**_r_* = 717.11	Mo *K*α radiation, λ = 0.71073 Å
Trigonal, *P*3	Cell parameters from 8040 reflections
*a* = 18.4876 (10) Å	θ = 2.8–32.1°
*c* = 11.5021 (7) Å	µ = 0.76 mm^−^^1^
*V* = 3404.6 (3) Å^3^	*T* = 180 K
*Z* = 6	Prism, dark green
*F*(000) = 2070	0.39 × 0.11 × 0.05 mm

### Data collection

**Table:**

Oxford Diffraction XCALIBUR diffractometer	4635 independent reflections
Radiation source: fine-focus sealed tube	2801 reflections with *I* > 2σ(*I*)
graphite	*R*_int_ = 0.063
Detector resolution: 8.2632 pixels mm^-1^	θ_max_ = 26.4°, θ_min_ = 2.8°
ω and φ scans	*h* = −23→23
Absorption correction: multi-scan (*CrysAlis RED*; Oxford Diffraction, 2008)	*k* = −23→23
*T*_min_ = 0.731, *T*_max_ = 1.000	*l* = −14→14
26629 measured reflections	

### Refinement

**Table:**

Refinement on *F*^2^	Primary atom site location: structure-invariant direct methods
Least-squares matrix: full	Secondary atom site location: difference Fourier map
*R*[*F*^2^ > 2σ(*F*^2^)] = 0.030	Hydrogen site location: inferred from neighbouring sites
*wR*(*F*^2^) = 0.068	H-atom parameters constrained
*S* = 0.97	*w* = 1/[σ^2^(*F*_o_^2^) + (0.0279*P*)^2^] where *P* = (*F*_o_^2^ + 2*F*_c_^2^)/3
4635 reflections	(Δ/σ)_max_ = 0.001
362 parameters	Δρ_max_ = 0.78 e Å^−^^3^
0 restraints	Δρ_min_ = −0.44 e Å^−^^3^

### Special details

**Table:**

Geometry. All esds (except the esd in the dihedral angle between two l.s. planes) are estimated using the full covariance matrix. The cell esds are taken into account individually in the estimation of esds in distances, angles and torsion angles; correlations between esds in cell parameters are only used when they are defined by crystal symmetry. An approximate (isotropic) treatment of cell esds is used for estimating esds involving l.s. planes.
Refinement. Refinement of *F*^2^ against ALL reflections. The weighted *R*-factor wR and goodness of fit *S* are based on *F*^2^, conventional *R*-factors *R* are based on *F*, with *F* set to zero for negative *F*^2^. The threshold expression of *F*^2^ > σ(*F*^2^) is used only for calculating *R*-factors(gt) etc. and is not relevant to the choice of reflections for refinement. *R*-factors based on *F*^2^ are statistically about twice as large as those based on *F*, and *R*- factors based on ALL data will be even larger.

### Fractional atomic coordinates and isotropic or equivalent isotropic displacement parameters (Å^2^)

**Table:**

	*x*	*y*	*z*	*U*_iso_*/*U*_eq_	
Mo1	1.0000	1.0000	0.22101 (5)	0.02614 (14)	
O11	0.92251 (14)	1.02746 (14)	0.12480 (19)	0.0300 (6)	
O12	0.89820 (15)	0.92784 (14)	0.3234 (2)	0.0328 (6)	
C11	0.8485 (2)	1.0084 (2)	0.1500 (3)	0.0292 (9)	
C12	0.8278 (2)	0.9228 (2)	0.3202 (3)	0.0334 (9)	
C13	0.8008 (2)	0.9608 (2)	0.2420 (3)	0.0346 (10)	
H13	0.7466	0.9537	0.2521	0.042*	
C111	0.8113 (3)	1.0438 (3)	0.0641 (4)	0.0412 (10)	
C121	0.7685 (3)	0.8663 (3)	0.4136 (3)	0.0429 (11)	
F11	0.84649 (19)	1.12517 (17)	0.0790 (2)	0.0781 (9)	
F12	0.82575 (17)	1.03301 (18)	−0.0431 (2)	0.0653 (8)	
F13	0.73139 (16)	1.01295 (19)	0.0767 (2)	0.0829 (10)	
F14	0.74487 (16)	0.78765 (16)	0.3919 (2)	0.0704 (8)	
F15	0.70096 (14)	0.87247 (17)	0.4230 (2)	0.0698 (9)	
F16	0.80526 (14)	0.88434 (15)	0.51746 (18)	0.0532 (7)	
Mo2	0.3333	0.6667	1.02042 (5)	0.03124 (15)	
O21	0.39312 (16)	0.77397 (15)	0.92317 (19)	0.0357 (6)	
O22	0.28812 (16)	0.72680 (17)	1.1206 (2)	0.0370 (6)	
C21	0.3944 (2)	0.8419 (2)	0.9427 (3)	0.0351 (10)	
C22	0.3029 (2)	0.8010 (3)	1.1092 (3)	0.0332 (10)	
C23	0.3536 (3)	0.8590 (2)	1.0281 (3)	0.0373 (10)	
H23	0.3606	0.9135	1.0316	0.045*	
C211	0.4503 (4)	0.9104 (3)	0.8578 (4)	0.0564 (12)	
C221	0.2561 (3)	0.8234 (3)	1.1961 (4)	0.0548 (13)	
F21	0.5295 (2)	0.93326 (19)	0.8780 (3)	0.0991 (11)	
F22	0.4346 (2)	0.88536 (15)	0.7498 (2)	0.0870 (10)	
F23	0.4454 (2)	0.97810 (17)	0.8698 (2)	0.0875 (10)	
F24	0.1770 (2)	0.7858 (2)	1.1699 (3)	0.1105 (13)	
F25	0.26262 (18)	0.80214 (18)	1.3017 (2)	0.0743 (9)	
F26	0.2806 (2)	0.9036 (2)	1.1958 (3)	0.1124 (13)	
Mo3	0.3333	0.6667	0.54730 (5)	0.03462 (16)	
O31	0.39271 (18)	0.77270 (15)	0.4469 (2)	0.0403 (6)	
O32	0.44118 (15)	0.71545 (18)	0.64445 (19)	0.0377 (6)	
C31	0.4665 (3)	0.8327 (3)	0.4578 (3)	0.0385 (10)	
C32	0.5086 (2)	0.7827 (3)	0.6246 (3)	0.0367 (10)	
C33	0.5252 (2)	0.8409 (2)	0.5390 (3)	0.0377 (10)	
H33	0.5793	0.8888	0.5356	0.045*	
C311	0.4891 (4)	0.8998 (3)	0.3686 (4)	0.0582 (14)	
C321	0.5779 (3)	0.7956 (4)	0.7077 (4)	0.0577 (13)	
F31	0.4518 (2)	0.94281 (18)	0.3915 (3)	0.0957 (11)	
F32	0.56998 (19)	0.95469 (18)	0.3661 (2)	0.0947 (11)	
F33	0.46553 (18)	0.86962 (17)	0.2642 (2)	0.0817 (10)	
F34	0.6111 (2)	0.7510 (3)	0.6743 (3)	0.1150 (13)	
F35	0.55263 (14)	0.7768 (2)	0.8139 (2)	0.0820 (9)	
F36	0.64080 (17)	0.8732 (2)	0.7069 (3)	0.0984 (12)	

### Atomic displacement parameters (Å^2^)

**Table:**

	*U*^11^	*U*^22^	*U*^33^	*U*^12^	*U*^13^	*U*^23^
Mo1	0.02183 (17)	0.02183 (17)	0.0348 (3)	0.01092 (9)	0.000	0.000
O11	0.0287 (15)	0.0307 (15)	0.0327 (15)	0.0163 (13)	0.0021 (12)	0.0054 (11)
O12	0.0271 (15)	0.0280 (15)	0.0375 (16)	0.0094 (12)	−0.0027 (12)	0.0045 (11)
C11	0.030 (2)	0.031 (2)	0.029 (2)	0.017 (2)	−0.0049 (19)	−0.0059 (18)
C12	0.028 (2)	0.030 (2)	0.032 (2)	0.007 (2)	−0.0054 (17)	−0.0056 (19)
C13	0.028 (2)	0.046 (3)	0.030 (2)	0.019 (2)	−0.0003 (19)	−0.001 (2)
C111	0.041 (3)	0.055 (3)	0.038 (3)	0.031 (3)	0.006 (2)	0.007 (2)
C121	0.035 (3)	0.051 (3)	0.032 (3)	0.013 (2)	−0.001 (2)	0.005 (2)
F11	0.112 (3)	0.058 (2)	0.089 (2)	0.061 (2)	−0.0238 (18)	−0.0026 (16)
F12	0.095 (2)	0.102 (2)	0.0347 (15)	0.0761 (19)	−0.0062 (14)	0.0026 (14)
F13	0.0444 (17)	0.129 (3)	0.089 (2)	0.0540 (18)	0.0083 (15)	0.0499 (19)
F14	0.072 (2)	0.0407 (17)	0.0615 (18)	0.0002 (15)	0.0110 (14)	0.0096 (14)
F15	0.0354 (14)	0.116 (3)	0.0551 (16)	0.0353 (15)	0.0171 (12)	0.0278 (15)
F16	0.0472 (14)	0.0699 (18)	0.0291 (13)	0.0192 (14)	−0.0013 (11)	0.0068 (12)
Mo2	0.0247 (2)	0.0247 (2)	0.0442 (4)	0.01237 (10)	0.000	0.000
O21	0.0316 (16)	0.0292 (15)	0.0412 (16)	0.0112 (13)	0.0095 (14)	0.0001 (11)
O22	0.0373 (17)	0.0328 (17)	0.0435 (16)	0.0195 (14)	0.0119 (14)	0.0073 (14)
C21	0.041 (3)	0.028 (2)	0.029 (2)	0.011 (2)	0.0001 (19)	−0.0001 (19)
C22	0.039 (2)	0.040 (3)	0.030 (2)	0.027 (2)	−0.0014 (19)	0.000 (2)
C23	0.053 (3)	0.032 (2)	0.033 (2)	0.026 (2)	0.003 (2)	−0.002 (2)
C211	0.068 (4)	0.038 (3)	0.044 (3)	0.013 (3)	0.020 (3)	0.010 (2)
C221	0.068 (4)	0.066 (3)	0.051 (3)	0.048 (3)	0.011 (3)	0.007 (3)
F21	0.066 (2)	0.067 (2)	0.120 (3)	−0.0003 (18)	0.031 (2)	0.0258 (19)
F22	0.141 (3)	0.0547 (16)	0.0318 (15)	0.024 (2)	0.0277 (18)	0.0060 (12)
F23	0.152 (3)	0.0401 (17)	0.0640 (19)	0.0431 (19)	0.0365 (18)	0.0197 (14)
F24	0.074 (2)	0.199 (4)	0.098 (2)	0.098 (3)	0.006 (2)	−0.024 (2)
F25	0.113 (2)	0.113 (2)	0.0348 (16)	0.086 (2)	0.0201 (15)	0.0130 (15)
F26	0.203 (4)	0.091 (2)	0.095 (2)	0.112 (3)	0.065 (2)	0.0199 (19)
Mo3	0.0314 (2)	0.0314 (2)	0.0411 (4)	0.01569 (11)	0.000	0.000
O31	0.0419 (18)	0.0353 (15)	0.0406 (16)	0.0170 (16)	−0.0086 (15)	0.0052 (12)
O32	0.0329 (15)	0.0398 (17)	0.0394 (16)	0.0175 (15)	0.0020 (12)	0.0113 (14)
C31	0.050 (3)	0.035 (3)	0.031 (3)	0.021 (2)	−0.001 (2)	0.001 (2)
C32	0.030 (2)	0.049 (3)	0.032 (2)	0.020 (2)	0.0040 (19)	0.007 (2)
C33	0.032 (2)	0.042 (3)	0.031 (2)	0.012 (2)	0.0044 (19)	0.005 (2)
C311	0.071 (4)	0.040 (3)	0.045 (3)	0.014 (3)	−0.012 (3)	0.007 (3)
C321	0.037 (3)	0.082 (4)	0.048 (3)	0.026 (3)	−0.001 (2)	0.016 (3)
F31	0.148 (3)	0.067 (2)	0.096 (2)	0.072 (2)	−0.003 (2)	0.0206 (17)
F32	0.079 (2)	0.068 (2)	0.087 (2)	0.0004 (19)	−0.0146 (18)	0.0430 (17)
F33	0.114 (3)	0.0654 (19)	0.0379 (16)	0.0244 (18)	−0.0140 (16)	0.0115 (14)
F34	0.097 (3)	0.183 (4)	0.117 (3)	0.108 (3)	−0.030 (2)	−0.012 (3)
F35	0.0481 (15)	0.124 (3)	0.0445 (16)	0.0212 (19)	−0.0042 (12)	0.0350 (19)
F36	0.0482 (18)	0.103 (3)	0.083 (2)	−0.0081 (19)	−0.0228 (16)	0.0361 (19)

### Geometric parameters (Å, °)

**Table:**

Mo1—O12	2.049 (2)	C23—H23	0.9500
Mo1—O11	2.064 (2)	C211—F23	1.306 (5)
O11—C11	1.265 (4)	C211—F22	1.306 (5)
O12—C12	1.259 (4)	C211—F21	1.325 (6)
C11—C13	1.377 (5)	C221—F25	1.300 (5)
C11—C111	1.524 (5)	C221—F24	1.303 (5)
C12—C13	1.378 (5)	C221—F26	1.314 (5)
C12—C121	1.517 (5)	Mo3—O31	2.057 (2)
C13—H13	0.9500	Mo3—O32	2.059 (2)
C111—F12	1.297 (4)	O31—C31	1.262 (4)
C111—F13	1.299 (4)	O32—C32	1.265 (4)
C111—F11	1.318 (5)	C31—C33	1.382 (5)
C121—F15	1.314 (4)	C31—C311	1.499 (6)
C121—F14	1.316 (5)	C32—C33	1.375 (5)
C121—F16	1.332 (4)	C32—C321	1.518 (5)
Mo2—O22	2.048 (2)	C33—H33	0.9500
Mo2—O21	2.053 (2)	C311—F33	1.303 (5)
O21—C21	1.265 (4)	C311—F31	1.314 (6)
O22—C22	1.265 (4)	C311—F32	1.323 (5)
C21—C23	1.368 (5)	C321—F35	1.292 (5)
C21—C211	1.522 (5)	C321—F34	1.308 (6)
C22—C23	1.375 (5)	C321—F36	1.320 (6)
C22—C221	1.510 (6)		
			
O12^i^—Mo1—O12	90.24 (9)	C21—C23—H23	118.4
O12—Mo1—O11^i^	88.97 (9)	C22—C23—H23	118.4
O12^i^—Mo1—O11	176.92 (10)	F23—C211—F22	109.3 (4)
O12—Mo1—O11	86.78 (9)	F23—C211—F21	105.7 (4)
O11^i^—Mo1—O11	93.96 (9)	F22—C211—F21	107.1 (4)
C11—O11—Mo1	126.6 (2)	F23—C211—C21	112.7 (4)
C12—O12—Mo1	127.6 (2)	F22—C211—C21	112.2 (4)
O11—C11—C13	127.7 (3)	F21—C211—C21	109.6 (4)
O11—C11—C111	112.9 (3)	F25—C221—F24	107.7 (4)
C13—C11—C111	119.4 (3)	F25—C221—F26	108.2 (4)
O12—C12—C13	127.4 (3)	F24—C221—F26	105.2 (4)
O12—C12—C121	113.2 (3)	F25—C221—C22	112.5 (4)
C13—C12—C121	119.5 (4)	F24—C221—C22	110.2 (4)
C11—C13—C12	123.2 (4)	F26—C221—C22	112.7 (4)
C11—C13—H13	118.4	O31^iii^—Mo3—O31	91.55 (10)
C12—C13—H13	118.4	O31—Mo3—O32^iii^	88.04 (10)
F12—C111—F13	108.3 (4)	O31^iii^—Mo3—O32	178.54 (11)
F12—C111—F11	106.1 (4)	O31—Mo3—O32	87.06 (10)
F13—C111—F11	106.9 (4)	O32—Mo3—O32^ii^	93.33 (9)
F12—C111—C11	112.2 (3)	C31—O31—Mo3	127.5 (2)
F13—C111—C11	113.3 (3)	C32—O32—Mo3	126.5 (2)
F11—C111—C11	109.6 (3)	O31—C31—C33	127.4 (4)
F15—C121—F14	107.9 (4)	O31—C31—C311	113.0 (4)
F15—C121—F16	107.0 (3)	C33—C31—C311	119.6 (4)
F14—C121—F16	107.0 (3)	O32—C32—C33	128.5 (4)
F15—C121—C12	112.9 (3)	O32—C32—C321	112.3 (4)
F14—C121—C12	110.7 (4)	C33—C32—C321	119.2 (4)
F16—C121—C12	111.1 (3)	C32—C33—C31	122.9 (4)
O22—Mo2—O22^ii^	91.41 (10)	C32—C33—H33	118.6
O22—Mo2—O21^iii^	88.26 (10)	C31—C33—H33	118.6
O22—Mo2—O21	87.18 (10)	F33—C311—F31	106.3 (4)
O21^iii^—Mo2—O21	93.14 (9)	F33—C311—F32	108.5 (4)
O22—Mo2—O21^ii^	178.54 (10)	F31—C311—F32	105.9 (4)
C21—O21—Mo2	126.6 (2)	F33—C311—C31	112.4 (4)
C22—O22—Mo2	127.2 (2)	F31—C311—C31	110.4 (4)
O21—C21—C23	128.2 (4)	F32—C311—C31	112.9 (4)
O21—C21—C211	112.3 (4)	F35—C321—F34	109.1 (5)
C23—C21—C211	119.5 (4)	F35—C321—F36	107.6 (4)
O22—C22—C23	127.6 (4)	F34—C321—F36	104.3 (4)
O22—C22—C221	112.7 (4)	F35—C321—C32	113.0 (4)
C23—C22—C221	119.7 (4)	F34—C321—C32	110.0 (4)
C21—C23—C22	123.1 (4)	F36—C321—C32	112.5 (4)

Symmetry codes: (i) −*y*+2, *x*−*y*+1, *z*; (ii) −*x*+*y*, −*x*+1, *z*; (iii) −*y*+1, *x*−*y*+1, *z*.

### Table 1 Comparison of some geometric parameters (Å, °) within the chelating acetonate units of related compounds

**Table:**

Complex	*M*—O (mean value)	*M*—O—*M* (mean value)	O···O
[Mo(hfacac)_3_]*^a^*	2.053 (4)	87.01 (9)	2.829 (4)
[Mo(acac)_3_]*^b^*	2.072 (3)	87.4 (1)	2.866 (3)
[Cr(hfacac)_3_]*^c^*	1.957 (1)	90.63 (5)	2.782 (2)
[Cr(hfacac)_3_]*^d^*	1.949 (5)	90.8 (2)	2.776 (7)

Notes: (*a*) This work; (*b*) Raston & White (1979); (*c*) Jessop *et al.* (2002); (*d*) Harada & Girolami (2007). acac is acetylacetonate and hfacac is 1,1,1,5,5,5-hexafluoroacetylacetonate.
